# Coronavirus disease 2019: achieving good mental health during social isolation

**DOI:** 10.1192/bjp.2020.91

**Published:** 2020-05-04

**Authors:** Rowan Diamond, John Willan

**Affiliations:** 1 Department of Psychiatry, University of Oxford, UK; 2 Wexham Park Hospital, Frimley Health Foundation NHS Trust; and Oxford University Hospitals NHS Foundation Trust, UK

**Keywords:** Psychosocial interventions, depressive disorders, information technologies, social deprivation, social functioning

## Abstract

The coronavirus disease 2019 pandemic has led to unprecedented disruption to the normal way of life for people around the globe. Social distancing, self-isolation or shielding have been strongly advised or mandated in most countries. We suggest evidence-based ways that people can maintain or even strengthen their mental health during this crisis.

Attempts to contain the spread of the coronavirus disease 2019 (COVID-19) pandemic have caused fundamental changes to the way of life of individuals around the world. Governments have encouraged or mandated the majority of their populations to stay at home wherever possible and practice social distancing. The UK Government has also required that the most vulnerable groups of individuals should shield themselves completely.

Governments have recognised that self-isolation has its own risks, including those of loneliness and deterioration of mental health, even in those without pre-existing mental health problems.^1^ Redundancy, furloughing or an inability to work, as well as the associated financial issues and changes in family dynamics, can exacerbate this problem. The consequences for people with severe mental health problems who are three times more likely to have a physical health problem than those in the general population are likely to be even more significant.^2^ Never has the connection between physical and mental health been so important or relevant.

In an attempt to improve personal well-being both for those with and without mental health problems, a set of evidence-based actions have previously been developed. The ‘Five Ways to Wellbeing’^3^ are a simple set of practical actions that can be performed daily to help achieve this. They are to learn, connect, take notice, give and be active.

We suggest ways in which all those who are self-isolating can attend to, or perhaps even improve, their mental and physical well-being under these most unusual of circumstances.

## Learn

The acquisition of new knowledge can give a sense of achievement and reward. Most magazines and newspapers will contain new information that may stimulate learning and offer opportunities for development of skills. For those with access to the internet and knowledge of how to use it, free teaching on a range of subject matters is easily accessible; for example, learning how to cook, play a musical instrument or put up shelves. Online libraries allow ongoing access to resources such as audiobooks, which, in the UK, are freely accessible with a library card.

## Connect

Meaningful interaction with others can promote self-worth and a sense of identity. Although the social restrictions brought about by COVID-19 might seem to reduce the possibility of regular contact with others, people are likely to have increased time for letter writing or speaking on the telephone. There are also opportunities to develop new and fulfilling ways of remote social interaction. The rapid development of new technology and its use as a communication tool has been received with mixed responses, with some expressing concern that it removes the genuine or real connection between people and reduces it to less meaningful connections. Without the ability to meet face to face and with increased free time, these new technologies may be embraced, allowing them to be used regularly, to build familiarity with the technology and to allow more meaningful communication. New applications and social network groups are rapidly being formed between friends, families, colleagues and neighbours, to support vulnerable individuals and help manage the new guidelines on isolation. Ironically, the physical isolation of whole communities to their homes may allow the opportunity for connections to form where previously they had not done so.

## Take notice

Mindfulness, or taking notice of the present moment, can improve mental well-being and may be a useful technique to help deal with anxiety during the COVID-19 pandemic. One benefit of reconnecting with our bodies and the sensations they experience, is that it can enable the mind to focus away from negative content (for example, anxiety, loneliness and worry) and toward more pleasurable sensations. Savouring these experiences can enhance the enjoyment of them. Examples of this might include taking notice of the colours in a sunset, the smell of baking, the sound of the rain on the roof, the feel of a soft rug underfoot or the taste of a nice drink. Sharing these simple everyday experiences with others, perhaps through virtual social networks, may enhance enjoyment further.

## Give

Some of the older people in this group will remember fondly the camaraderie of war time and the post-war period, during which the act of giving led to a strengthening of bonds within a community. This current opportunity to pull together in the face of adversity and contribute toward a greater goal can give a similar sense of purpose, which can be experienced by everyone. Giving may seem more difficult to achieve when one is isolated; however, there are many different ways it can be achieved. It is possible to connect with isolated and potentially lonely people in the community, or more widely, by volunteering time or skills. This can be offered from home, perhaps by volunteering for pre-existing phone lines for elderly people (for example, The Silver Line, a telephone support scheme for older and more isolated individuals; see https://www.thesilverline.org.uk/). Some people may have skills in making things that could be sold to raise money for charity; others may have the resources and ability to use their garden to produce food or flowers that could be delivered by others to their neighbours. It is possible that there may be difficulties in obtaining certain foods, and sharing these will help to support self-worth.

## Be active

Physical activity has been described by the Academy of Medical Sciences as a ‘miracle cure’, with impressive evidence of benefits to body and mind.^4^ Physical activity is safe and beneficial for almost everyone and any level of physical activity is better than none.^4^ Indeed, reducing the duration of time spent sitting, even if just standing up, is linked to improved physical health outcomes independently of how much exercise people do.^5^ During periods of COVID-19 lockdown, the usual opportunities for physical activity are much reduced, although many have taken up running to maximise their allowed time outside the house. People may be confined to small living quarters with no outdoor space, or have no opportunity to exercise with others or attend regular exercise facilities. However, exercising at home is possible for people with a range of abilities and conditions, and can be guided from readily available DVDs. There are also opportunities for people to join virtual live classes via platforms such as YouTube. This may provide a perfect opportunity for people who previously were not able to access such classes, perhaps because of mental or physical health problems or accessibility issues. It is recommended that people unfamiliar with regular exercise and those with underlying health conditions should ‘start slow and build up’ to avoid injury^4^ or exacerbation of symptoms. Even light physical activity has been shown to have positive health and well-being benefits in those with limited prior experience of exercise. To avoid sitting too much, people may choose certain times (for example, advert breaks) when they will get up and move, even if only to stand up and sit down again.

## Building a routine

Having a regular routine is important for physical and mental well-being and can help to ease the disturbance caused by the loss of the usual daily structures of work and school. Finding appealing activities that fit into each of these five categories are likely to be helpful in maintaining mental health and contributing toward better physical health. Using these activities to build some regular routines into the day may be particularly helpful when the usual daily timetable of activities has been disrupted. Trying to maintain normal sleep/wake cycles will help preserve good mental health, and significant increased use of stimulants, such as coffee, or sedatives, such as alcohol, should be avoided.

## Conclusion

Dame Vera Lynn, at 103 years of age, said of this pandemic that ‘even if we're isolated in person we can still be united in spirit’, and the sense of purpose that may be engendered in self-isolation may paradoxically lead to improvements in the mental health of some individuals who may otherwise feel that they have lost their role in society. Those for whom isolation is a new challenge should be encouraged to view opportunities to change the way they live their lives. Those for whom isolation is sadly the norm, including some with severe mental illness, could benefit from the myriad new initiatives arising in these unusual times.

This is a time of enormous disruption to routine, with the possibility of social isolation and mental health deterioration. We hope that these suggestions, combined with new opportunities, may enhance well-being, potentially even beyond pre-COVID-19 levels.

## Supplementary material

For supplementary material accompanying this paper visit http://dx.doi.org/10.1192/bjp.2020.91. click here to view supplementary material
